# Legal Notes for Institutional Workers

**Published:** 1917-01-20

**Authors:** 


### LEGAL NOTES FOR INSTITUTIONAL WORKERS.

### Suicide after Injury.—Compensation.

It has already been decided that where a workman has suffered some organic or structural injury to the brain, due to accident in the course of his employment, and in consequence becomes insane and commits suicide, his dependants are entitled to compensation under the statute, the death being considered in law a direct result of the injury, but in a recent case (*Withers* v. *London, Brighton and South Coast Railway Company*, 141 L.T.J. 272) the Court of Appeal refused to entertain an ingenious extension of that principle. It was maintained for the claimants that whenever an accident occurs to a workman which involves, as so many accidents do, depression of spirits—particularly depression of spirits in the case of a workman who has been leading an active life—and he commits suicide, the same no less than neurasthenia was directly attributable to the accident. The Court did not give effect to that specious plea, and, though sympathising with the dependants, dismissed their claim.

### Nurse's Street Accident and Compensation.

An interesting case affecting a nurse recently arose under the Workmen's Compensation Act. There have been conflicting decisions on the question whether those meeting with street accidents are entitled to the benefits of the statute. Such a risk is, of course, one to which any pedestrian is liable, and cannot be said to specially "arise out" of any employment so as to entitle the employee to compensation. There have been cases, however, in which the workman was found entitled to compensation in respect of street accidents on the theory that by his employment he was more especially exposed to these dangers than other people were. Thus a commercial traveller going about a town soliciting orders and run over by a vehicle in the course of his rounds has been held entitled to an award. On the other hand, a solicitor's clerk cycling, as he was accustomed to do, from his employer's office to attend Quarter Sessions, and sustaining injury through collision with a motor-car, was found not entitled to compensation; and similarly a ship's captain who had been ashore seeing his owners, and in the course of returning to his ship slipped on a piece of banana skin and was badly injured in consequence, was also held to have no claim under the Act, the reason being that in both these cases the resulting injuries were not attributable to the employment at all.

But now the case of *Ince* v. *Reigate Education Committee*, 141 L.T.J. 251, has brought things to a very fine point indeed. The applicant was a professional nurse engaged by the Education Committee as visiting nurse for their district, which was a residential rural locality. Her duties were to visit children as directed by the doctor, and to inspect schools. She was told to ride a bicycle, and the respondents hired one for her. She had to travel six to ten miles every day, and the number of cases that she visited averaged 120 per month. On the occasion in question this nurse left a house where she had been visiting a child, and was bicycling along the road when, owing to her cycle skidding when she was passing a cart, she was knocked against the vehicle and sustained serious injuries. Did this accident arise out of the applicant's employment The County Court Judge decided that the accident did so arise, and that on two grounds—first, that the employment compelled the applicant to do her work on a bicycle; and, secondly, that the risk in her case was greater owing to the nature of her employment than in the case of an ordinary cyclist. This decision, however, has been reversed by the Court of Appeal on the grounds that a person cycling on an ordinary road was doing what was safe, that the risk of the road or street did not entitle the pedestrian to compensation, and a bicyclist could not be in a better position; also that riding a bicycle as the applicant had to do did not expose her to any greater risk than ordinary cyclists ran, and that therefore she was not entitled to any statutory payment.

### Unsound Food.—Appeal against Conviction.

The case of *Webb* v. *Baker*, 61 S.J. 72, should be noted by practitioners concerned with Public Health prosecutions. It was a prosecution of a provision contractor who had supplied to a public institution a quantity of rabbits alleged to be unfit for human food. He was convicted before the Justices, and brought this appeal on the ground that, in the sense of the statute, he had not deposited the rabbits for sale, and that at the time they were seized they were not in his possession. The Court sustained the second ground of appeal and quashed the conviction. In this case we wish to call attention to the concise and neat interpretation of the statute given in the following sentence from the opinion of the Lord Chief Justice: "If we come to the conclusion that the rabbits were not deposited by the appellant for the purposes of sale within the meaning of the statute, the conviction cannot stand, notwithstanding that the rabbits might be in the possession of the appellant at the material time; and equally if we hold that the rabbits were deposited for the purpose of sale by the appellant, but were not in his possession at the material time, the conviction cannot stand."

